# Hernia Following Rectus Sheath Hematoma

**DOI:** 10.7759/cureus.28795

**Published:** 2022-09-05

**Authors:** Adam Dulberger, Mitchell Streiff, Scott D Myers, Christopher S Sanders

**Affiliations:** 1 Department of Diagnostic Radiology, David Grant Medical Center, Fairfield, USA; 2 Department of Radiology, Ponce Health Sciences University, Ponce, USA; 3 Department of Interventional Radiology, David Grant Medical Center, Fairfield, USA

**Keywords:** rectus sheath hematoma, rectus hematoma, rectus sheath hernia, posterior rectus sheath hematoma, abdominal wall hernia

## Abstract

Rectus sheath hematomas (RSH) are increasing in prevalence, presumably correlating with increased use of anticoagulation medications and an aging population. Comorbidities such as blood dyscrasias, atherosclerosis, and hypertension are associated with an increased risk of developing an RSH. Iatrogenic origin of RSH, secondary to treatment of various abdominal pathologies, is not uncommon. Due to its exceptionally non-specific array of clinical signs and symptoms, RSH can be challenging to diagnose in the clinical setting without the aid of radiological images. Abdominal computed tomography (CT) is generally the modality of choice through which the RSH can be successfully identified and characterized. CT imaging can play an important role in the planning of RSH management, as effective management varies depending on the size and position of the RSH. Recurrent bleeding, hypovolemic shock, abdominal compartment syndrome, myonecrosis, and infection have been traditionally considered as the more prominent complications of RSH. However, with more cases occurring, more complications are being described in the literature. The following case presents a previously unreported complication of RSH, that of bowel herniation into a potential space created by a previously treated RSH.

## Introduction

Rectus sheath hematomas (RSH), most often the result of bleeding from an epigastric artery or one of its branches within the rectus sheath, are increasing in frequency and severity [1]. An aging population and more widespread use of anticoagulation medications correlate strongly with the rise in incidence of RSH. Patients of advanced age, with history of anticoagulation use, blood dyscrasias, atherosclerosis, hypertension, coughing, and/or pregnancy are at increased risk for developing RSH [2,3]. Iatrogenic causes of RSH are also relatively common and can include amniocentesis, paracentesis, abdominal surgery, abdominal wall injections, or even acupuncture [1]. Patients with RSH most often present with vague abdominal pain, a symptom obviously shared with numerous other pathologies, making the initial diagnosis of RSH a challenging one. Other symptoms associated with RSH include nausea, vomiting, fever, syncope, and urinary retention [3]. Given the frustratingly non-specific presentation of RSH, the list of differential diagnoses is often extensive, to include other abdominal pathologies such as appendicitis, diverticulitis, colitis, ovarian abscess, or placental abruption [1,2]. Due to the lack of specific symptomatology in RSH, radiological imaging can play a paramount role in a timely diagnosis. CT imaging, preferably done with IV contrast, can provide accurate identification and localization of an RSH [1,2,4]. Once correctly identified, management of RSH is widely variable, with many cases warranting only conservative management, while others require swift intervention to ligate or embolize an actively bleeding artery. Regardless of severity, a pause of anticoagulation medication is most often appropriate to avoid worsening of the RSH. Complications of RSH are generally rare but can be potentially life-threatening in the case of abdominal compartment syndrome, myonecrosis, or infection [1,2]. In this case we describe a seemingly rare complication of RSH, that of abdominal hernia into the space created by an RSH suffered years ago. 

## Case presentation

**Figure 1:**
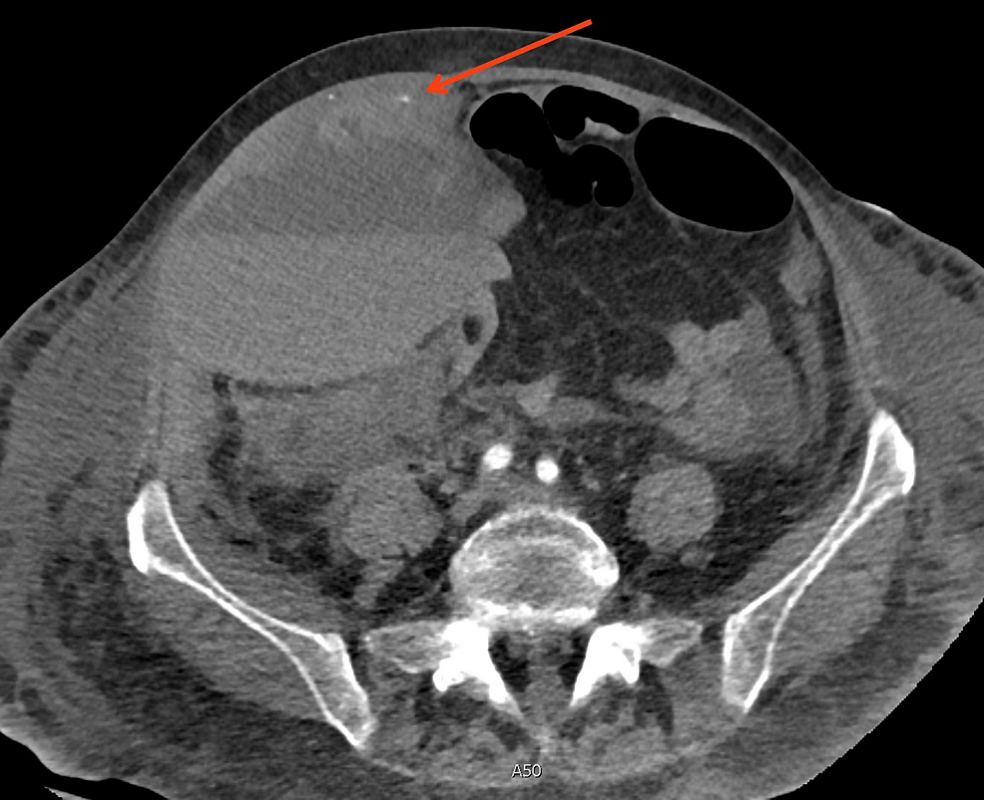
Active extravasation of the right inferior epigastric artery leading to a rectus sheath hematoma

Our patient is a 71-year-old female with chronic lymphocytic leukemia on anticoagulation for a history of deep vein thrombosis. She was admitted to the hospital for tachycardia, weakness and diarrhea, and ultimately diagnosed with Clostridium difficile colitis. Several days into her admission she developed right lower quadrant (RLQ) abdominal pain that was initially believed to be due to the colitis. However, over several days her pain worsened and she became hemodynamically unstable, requiring blood transfusions. Contrast enhanced CT demonstrated an RSH with active extravasation from the right inferior epigastric artery (Figure 1).

**Figure 2:**
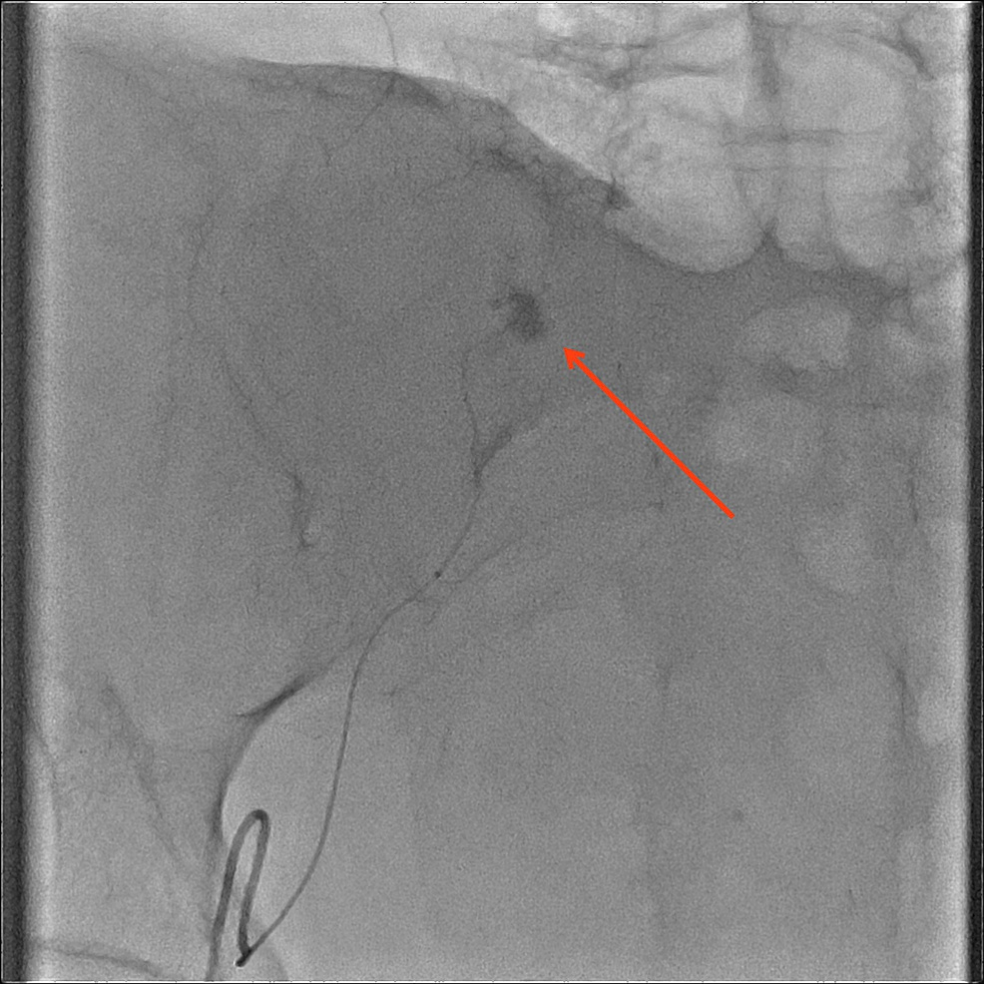
Inferior epigastric artery demonstrating active extravasation on angiography

**Figure 3:**
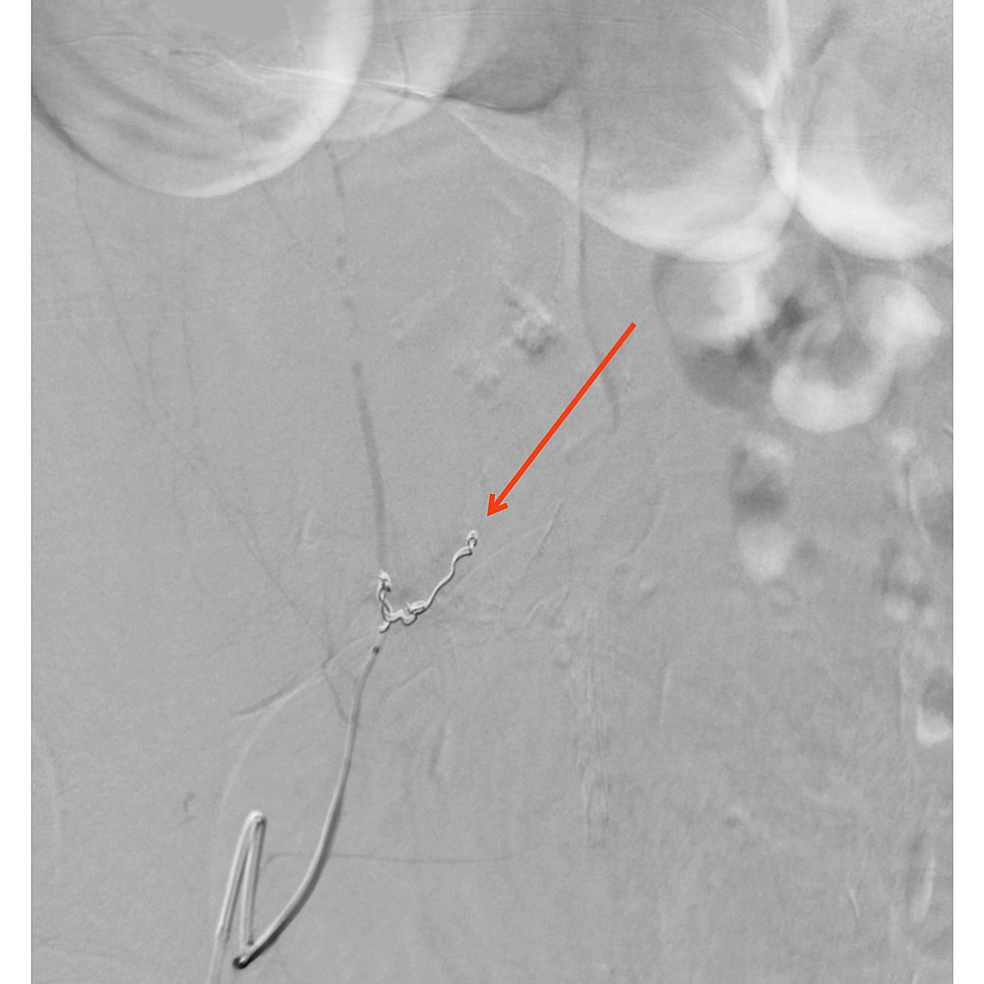
Appearance following embolization performed by Interventional Radiology

Providers initiated medical management, hoping the bleeding would subside. Unfortunately, the bleeding continued despite medical management and the team consulted Interventional Radiology (IR). IR decided to perform an inferior epigastric embolization procedure, after which the patient stabilized (Figures 2, 3). 

**Figure 4:**
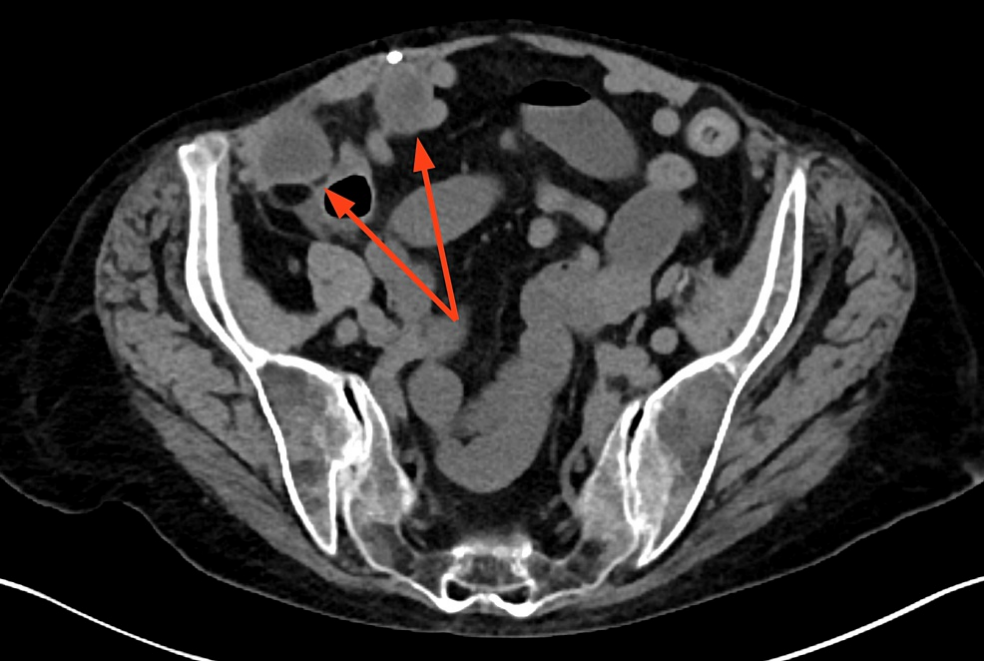
Herniation of bowel into space created by previous rectus sheath hematoma (axial)

**Figure 5:**
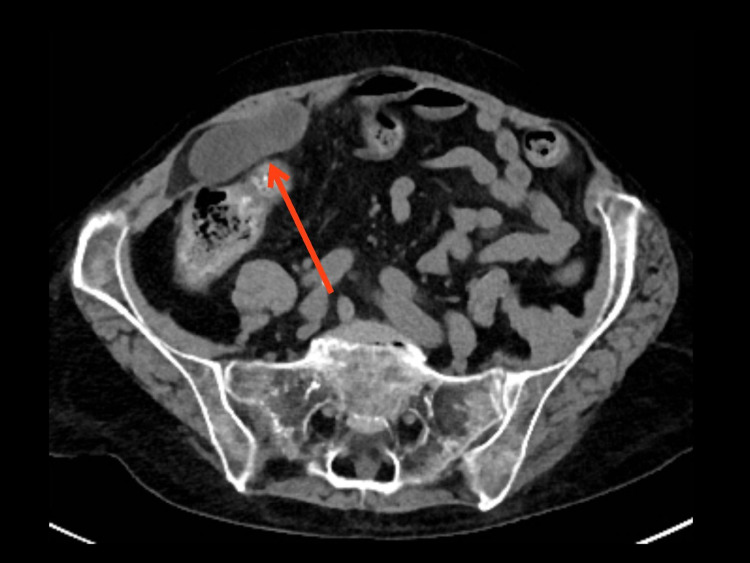
Herniation of bowel into space created by previous rectus sheath hematoma (axial)

**Figure 6:**
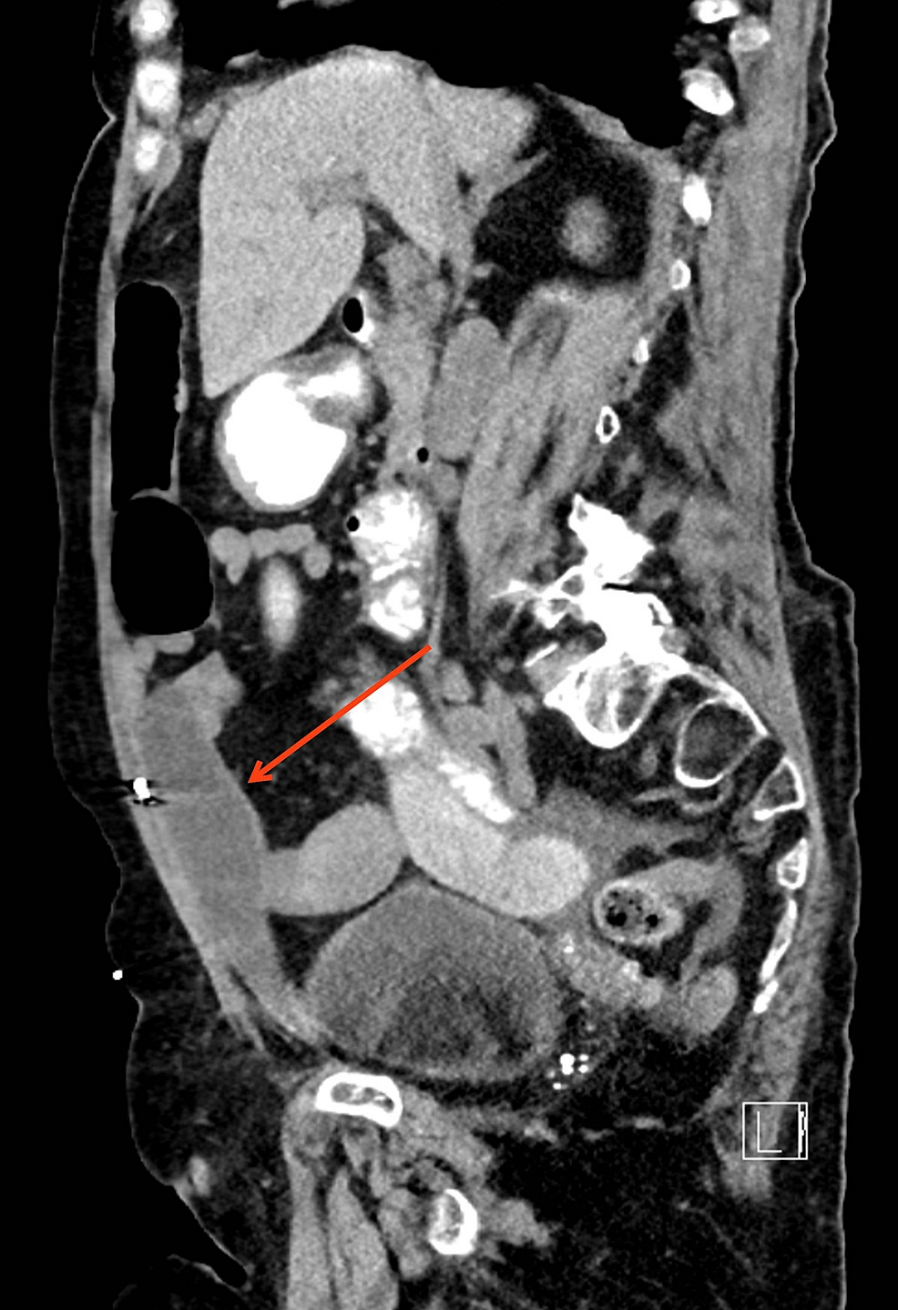
Herniation of bowel into space created by previous rectus sheath hematoma (sagittal)

**Figure 7:**
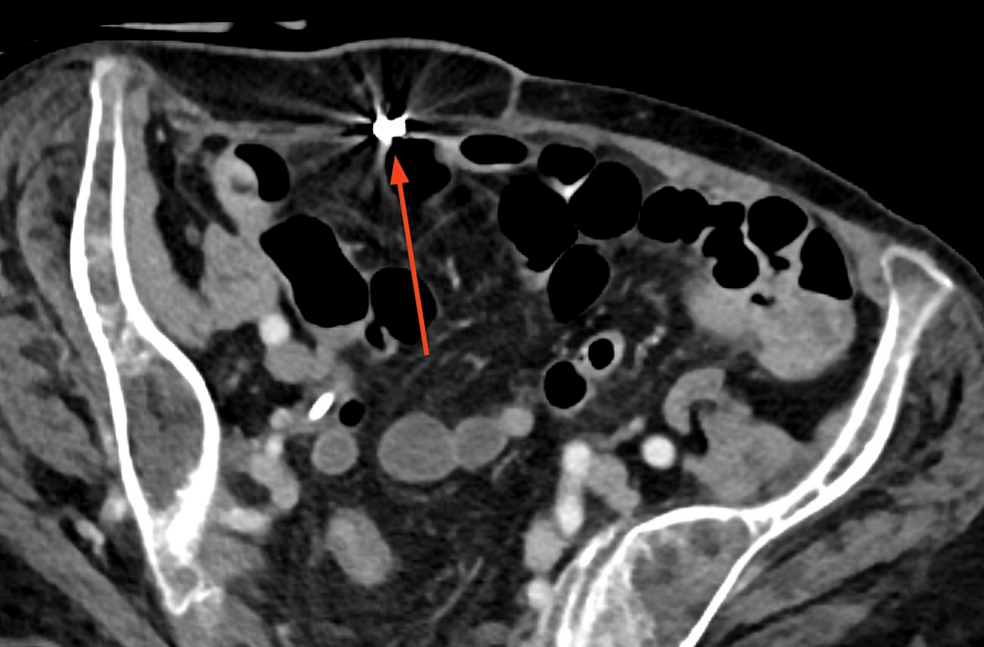
CT following surgical mesh repair of the hernia

Two years later she again developed RLQ pain, and was found to have a small bowel hernia that entered the space created by the prior RSH (Figures 4-6). Mesh repair was subsequently performed (Figure 7).

## Discussion

RSHs are increasing in frequency and severity, correlating with an aging population and greater use of systemic anticoagulation. As a consequence, complications from RSH will also increase, including more rare complications such as hernia into the resultant potential space [1]. 

The most common presenting symptom of RSH is abdominal pain. While pain may be focal or diffuse, it has been reported that diffuse pain is more frequent for RSHs greater than 3-5 cm in size [1]. Physical examination can demonstrate ecchymosis, tenderness to palpation, a palpable abdominal mass, or Grey-Turner’s sign [5]. Patients may develop hemodynamic instability indicated by tachycardia and hypotension, with declining hematocrit levels [1,2,5]. Given its non-specific presentation, RSH can mimic a wide array of other more common pathologies, making RSH a challenging initial diagnosis. 

RSH generally develops due to tearing of an epigastric artery or one of its branches within the rectus sheath. RSHs are more common below the arcuate line for several reasons. Below the arcuate line, the posterior fascia is less robust, as the internal oblique and transversus abdominis aponeuroses of the rectus sheath instead pass anteriorly to the rectus abdominis muscle, leaving only the transversalis fascia posteriorly. Additionally, there is the potential for communication between the inferior retrorectus space and the prevesicular space of Retzius, which creates a natural dissection plane [2,5]. Thirdly, contraction of the rectus abdominus mainly lengthens the lower half of the muscle, which exposes the branches of the inferior epigastric vessels to shearing forces [2]. 

CT is the modality of choice for detection of RSH and its complications [1,2,5]. Increased specificity is achieved using intravenous contrast, sometimes locating active bleeding. Non-visualization of active extravasation on CT should not prevent angiographic evaluation if clinical suspicion for ongoing bleeding is high [6]. In the case of an acute RSH, CT will likely show a hyperdense mass posterior to the rectus abdominis muscle with ipsilateral anterolateral muscle enlargement. Chronic RSH will appear differently on CT, generally manifesting with an isodense or hypodense mass relative to the surrounding tissue [7]. If CT is not available or feasible due to the patient’s state of health, ultrasound can be used to provide visualization of an RSH. Proper ultrasound technique can yield images showing a sonolucent mass that is spindle-shaped on sagittal sections and ovoid-shaped on coronal sections, suggestive of an RSH [8]. While rarely necessary, MRI imaging can also be used. It is most useful in the case of chronic RSH where it will show high signal intensity on both T1 and T2 weighted images in the anatomical area of interest [9]. 

Treatment depends on presentation, anticoagulation status, and hemodynamics. Because many mild RSHs are self-limited, supportive treatment with pain management, limited activity, and anticoagulation discontinuation can often lead to resolution [1,2]. Additionally, controlling risk factors such as hypertension can decrease the chance of recurrence [5]. Hemodynamically significant RSHs often require treatment with either surgical ligation of the bleeding vessel or percutaneous embolization [1]. Currently there are no clinical trials that demonstrate superiority of surgery over embolization [2]. However, surgical ligation has been associated with significant morbidity and mortality in patients with multiple co-morbidities and advanced age [10]. Trans-catheter arterial embolization is now considered first-line therapy due to its decreased risk profile and high technical success rate [1]. Angiography and arterial embolization have proven safe and effective in patients with large hematomas and hemodynamic compromise [2]. Ultimately, proper management will largely depend on the clinical status of the patient and the indications of radiological imaging.

Regardless of whether conservative or invasive management is employed, clinicians should consider sequelae of RSH when a patient with a history of RSH presents with abdominal pain. While not likely, such abdominal pain could be due to bowel herniation into the space created by a previous RSH, as was seen in this case. Rectus sheath hernia is a very rare entity, with only a few published cases. Most occur as the result of trauma or surgical weakening, with spontaneous cases much less common [11]. It is our hypothesis that a previous occurrence of RSH may weaken the fascia and predispose to hernia formation. Most of these hernias occur in a supraumbilical location, superior to the arcuate line, in contrast to our case [12]. Previously reported cases of rectus sheath hernias have largely been treated surgically, with techniques similar to other types of ventral hernias [11]. In our case laparoscopic mesh repair was performed, without known hernia recurrence.

## Conclusions

Although more common complications, such as recurrent bleeding, infection, or the rarer complication of abdominal compartment syndrome are well known, the delayed complication of abdominal hernia should be considered in patients with abdominal pain and a history of RSH. A review of the literature did not reveal small bowel herniation into the space created by the RSH as a complication. Quality abdominal imaging is the key to ensuring proper diagnosis and management in these individuals. Our hope is to raise awareness of this rare complication. Given that RSH is increasing in prevalence, it stands to reason that this hernia may occur more frequently in this demographic and should be considered highly on the list of differential diagnoses in a patient with abdominal pain and a history of RSH. 
